# Accelerating neurotechnology development using an Agile methodology

**DOI:** 10.3389/fnins.2024.1328540

**Published:** 2024-02-16

**Authors:** Anil Kumar Thota, Ranu Jung

**Affiliations:** ^1^Adaptive Neural Systems Group, The Institute for Integrative and Innovative Research, University of Arkansas, Fayetteville, AR, United States; ^2^Biomedical Engineering Department, University of Arkansas, Fayetteville, AR, United States

**Keywords:** medical device development, design controls, verification and validation, waterfall, Agile, neurotechnology, peripheral neural interfaces

## Abstract

Novel bioelectronic medical devices that target neural control of visceral organs (e.g., liver, gut, spleen) or inflammatory reflex pathways are innovative class III medical devices like implantable cardiac pacemakers that are lifesaving and life-sustaining medical devices. Bringing innovative neurotechnologies early into the market and the hands of treatment providers would benefit a large population of patients inflicted with autonomic and chronic immune disorders. Medical device manufacturers and software developers widely use the Waterfall methodology to implement design controls through verification and validation. In the Waterfall methodology, after identifying user needs, a functional unit is fabricated following the verification loop (design, build, and verify) and then validated against user needs. Considerable time can lapse in building, verifying, and validating the product because this methodology has limitations for adjusting to unanticipated changes. The time lost in device development can cause significant delays in final production, increase costs, and may even result in the abandonment of the device development. Software developers have successfully implemented an Agile methodology that overcomes these limitations in developing medical software. However, Agile methodology is not routinely used to develop medical devices with implantable hardware because of the increased regulatory burden of the need to conduct animal and human studies. Here, we provide the pros and cons of the Waterfall methodology and make a case for adopting the Agile methodology in developing medical devices with physical components. We utilize a peripheral nerve interface as an example device to illustrate the use of the Agile approach to develop neurotechnologies.

## 1 Introduction

Healthcare professionals routinely use various medical devices to save, support, and sustain patients’ lives. The United States of America Food and Drug Administration (FDA) and other international regulatory institutions provide guidance documents to medical device industry professionals for developing safe and effective medical devices and approve them for use by persons in need. Traditional medical devices have physical (electronics and/or mechanical) and sometimes also software components [referred to as “software in medical devices (SiMD)” (FDA, 2020)]. Recent advances in digital health have created a new device category, “software as a medical device (SaMD)” (FDA, 2018b), which works independently without the need for physical components (e.g., software that extracts diagnostic information from X-rays).

**Figure 1:**
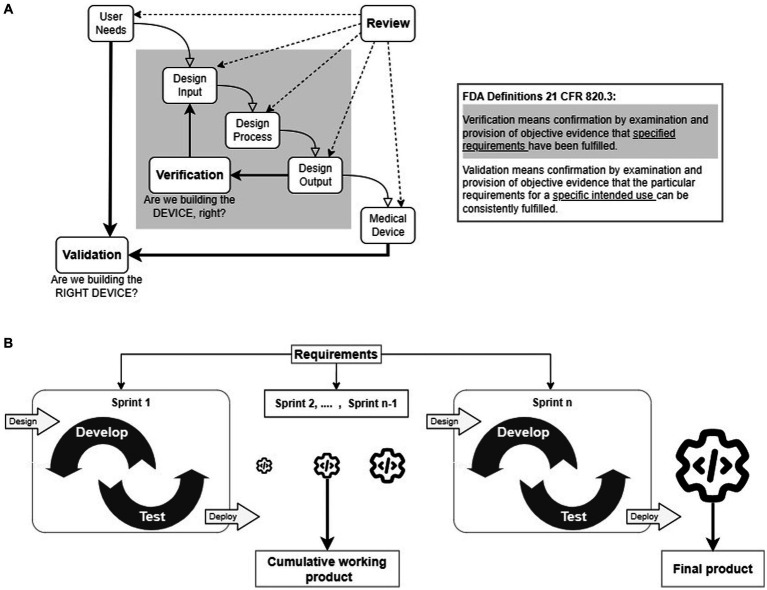
**(A)** Waterfall design control process. The waterfall methodology follows a step-by-step linear progression of logical sequences of design control processes. The *Design Verification* process, highlighted in gray, is an iterative process to confirm that the *Design Inputs* match the *Design Outputs*. *Design Validation* process is also an iterative process to prove that the device’s intended purpose is met by evaluating *User Needs*. The inset provides FDA-specified definitions of *verification* and *validation* processes. **(B)** The Agile framework. The Agile frameworks were developed from the Agile manifesto, which gives importance to flexibility to adapt to changes and quick delivery of the functional product for deployment. The primary concept of an Agile framework is to divide the project into small functional units (referred to as *sprints*) for development and testing. Each successful *sprint* enables the deployment of working software products. The final product is the summation of all the working software products.

The Waterfall methodology has been considered a *de facto* method for developing medical devices (hardware devices, SiMD, and SaMD) among established medical manufacturers. It follows sequential processes with strictly defined milestones (Figure 1A) with clear decision points for moving to the next steps. It is popular not only because the FDA suggested it in their guidance document but also because it provides a concise pathway for documenting the quality of the device design to ensure the safety and efficacy of the product. However, one of the primary criticisms of the Waterfall methodology is that the entire development process is too strict and has little room for adapting to unanticipated changes, which causes significant delays (Lee et al., 2006; Antonini et al., 2021; Slattery et al., 2022). To address such delays, the developers of SiMDs and SaMDs have adopted Agile, V-model, and other lean methodologies (Lee et al., 2006; Rottier and Rodrigues, 2008; Antonini et al., 2021; ScaledAgile, 2022; Slattery et al., 2022). In the Agile methodology (Figure 1B), the product is developed in increments, called *Sprints*, to produce testable and deployable products by allowing the requirements to evolve during the development to reduce delays.

The Agile inspired “Crisis-Responsive Framework” was developed during the COVID-19 pandemic to tackle the global scarcity crisis of personal protective equipment (PPE) by accelerating the production using unconventional sources for supply, engaging stakeholders throughout the development process, and modifying verification and validation processes (Antonini et al., 2021). Although this framework was successful in developing PPE, a Class I device, adopting Agile in developing high-risk (Class II/III) medical devices is challenging. One of the limiting factors is the need for preclinical and clinical validation studies to obtain regulatory approval (Famm et al., 2013; McHugh et al., 2013; Jung et al., 2017; Horn et al., 2019; Pavlov and Tracey, 2019; Cho et al., 2020; Larson and Meng, 2020; Magisetty and Park, 2022). These studies are time-consuming; hence, delivering a functional product quickly to test is challenging.

This review provides salient aspects and shortcomings of the Waterfall and Agile methodologies. It may spur a dialog among academic researchers and business leaders in neurotechnology development on the applicability and usefulness of Agile Frameworks. We illustrate the use of the Agile methodology as an alternative to the established Waterfall methodology using the development of electrodes for peripheral nerve interfaces (PNI) as an example.

## 2 Waterfall methodology

The FDA provides detailed regulations (*21 CFR 800–898*) and guidelines for developing, prescribing, and using medical devices (FDA, 2018a). The Design Controls (21CFR 820.30) are crucial for ensuring the quality of medical devices and user safety under quality management system regulations. The FDA released the “Design Control Guidance for Medical Device Manufacturers” guidance document, which introduced the use of design controls through the Waterfall methodology (FDA, 1998; Figure 1A). The Waterfall methodology follows a logical sequence (*User Needs*, *Design Input*, *Design Process,* and *Design Output*) followed by *Design Verification* and *Design Validation* iterative processes (FDA, 1998; Fry et al., 2016). Throughout the device development cycle, reviews are regularly conducted to compile the comprehensive documentation necessary for FDA approval.

Compiling a comprehensive list of *User Needs* at the beginning of the development process is critical for successful medical device development and deployment. To achieve this, multiple interviews and reviews are conducted to gather input from all the stakeholders (patients, doctors, device manufacturers, and others). The *User Needs* include intended uses of the device (primary purpose), indications for use (medical conditions aimed to diagnose, treat, or cure), and other end-user needs, such as operator and environmental needs.

After the development team approves the *User Needs* list, the *Design Inputs* are defined from the *User Needs* and are translated to *Design Specifications*. The *Design Specifications* are reviewed and approved to ensure that the specifications can be tested, measured, or observed.

In the *Design Process,* the approved *Design Specifications* are used to develop detailed documentation of product drawings, tools used, and instructions to produce tangible products (physical devices or computer models/programs). The design reviews are conducted and approved to ensure the proposed tools can produce the designed product. *Design* using the design documents developed in the *Design Process*.

The iterative *Design Verification* process ensures that the *Design Outputs* meet the *Design Inputs,* aka *Design Specifications* (see: gray shaded inner loop in Figure 1A). To achieve this, testing protocols are written for each *Design Specification,* and tests are run to compare with the corresponding *Design Output*. The design refinement continues iteratively until the *Design Outputs* meet the *Design Specifications.* At the end of each iteration, *Design Reviews* are conducted to monitor the quality of the designs and the ability of the designs to produce products to the specifications. A fully verified device is produced for *Design Validation*.

The iterative *Design Validation* process is the most critical development process (see: outer loop in Figure 1A). It ensures that the verified medical device meets all the defined *User Need*s, and is safe and effective for use. This process also continues until the finished product satisfies all the *User Needs* and may require conducting preclinical or clinical studies.

## 3 Need for an alternative device design control methodology

The primary reason for significant delays in utilizing the Waterfall method (FDA, 1998) is the need to make unanticipated changes during validation studies (Rottier and Rodrigues, 2008; Stare, 2014; Papadakis and Tsironis, 2020; Salisbury, 2021; Slattery et al., 2022). The unanticipated changes in *User Needs* and *Design Specifications* can cause significant delays in the development of the device, can become expensive, and could entirely stop the development process. The compiled comprehensive list of *User Needs* at the beginning of the development process may be incomplete or have incorrect entries due to misinterpreting stakeholders’ responses or other constraints in engaging various stakeholder groups. This leads to identifying new *User Needs* at later stages of device development, causing significant delays. Inadequate engagement with various stakeholders, insufficient literature review, or limited data from pilot studies may lead to errors in translating *User Needs* into *Design Specifications,* resulting in flaws in defining each design specification’s accuracy, precision, and tolerance metrics. This may, in turn, lead to failure in design verification or require an increased number of verification loops, causing delays and or depletion of resources.

Changes during medical device development are inevitable. Hence, there is a need for an alternative design control methodology that can adapt to the unanticipated changes during the product development process.

## 4 Agile methodology as an alternative

A group of software developers who found the Waterfall methodology highly ineffective authored the “Agile Manifesto” (Beck et al., 2001) with four values and twelve principles. Based on these values and principles, software developers have created multiple Agile frameworks such as Scrum, Extreme Programming, and Kanban (Stare, 2014; Theobald et al., 2019; Reiff and Schlegel, 2022). These Agile frameworks promote flexibility in following predefined processes, delivering products quickly, collaborating with end-users for validation tests, and in adjusting plans in response to changing requirements (Antonini et al., 2021). In Agile (Figure 1B), the project is divided into short, repeatable phases (Rottier and Rodrigues, 2008; Theobald et al., 2019; Birk, 2021; Holden et al., 2021; Reiff and Schlegel, 2022; ScrumAlliance, 2023), often referred to as *Sprints*. Each successful *Sprint* incorporates one or more requirements to build and evaluate a functional unit. The outcomes of the first *Sprint* are integrated into subsequent *Sprints* to develop an incrementally improved functional unit until the completion of the project. Thus, the effect of unanticipated changes in *User Needs* and *Design Specifications* can be mitigated by adopting the Agile methodology.

## 5 Barriers to adopting Agile for traditional medical devices

The Agile process, as used for general software development, does not meet the needs for the development of medical device software, which includes extensive planning for use, assuring traceability of requirements, rigorous documentation of the development process, and ability to meet regulatory compliance. However, some developers of SiMD and SaMD have overcome the above-mentioned operational barriers and are either already using or actively pursuing the Agile methodology (Birk, 2021) for developing medical device software to reduce time-to-market (Antonini et al., 2021; Salisbury, 2021). The authors of the Agile-inspired tailored “Crisis Crisis-Responsive Framework” described two significant Barriers to developing Class I traditional medical devices with physical components: increased verification and validation tasks, including biocompatibility testing, because at least one physical component (structural, mechanical, electronic, or a combination thereof) is in contact with living tissue, and extensive safety and efficacy documentation for regulatory approval (Antonini et al., 2021).

## 6 Adoption of the Agile methodology for developing peripheral nerve interfaces

Recent innovations in computer-aided programs for developing designs, finite element analysis for testing designs in realistic simulated conditions, and 3D printing technology offer opportunities for accelerating the development of devices with physical components utilizing the Agile process (FDA, 2017; Milovanović et al., 2019; Ghilan et al., 2020; Al-Dulimi et al., 2021; Calvo-Haro et al., 2021; Kumar et al., 2021; Algorri et al., 2022). Here, we use a peripheral nerve interface (PNI) design as an exemplar of the Agile methodology for medical hardware device development. An Agile methodology may accelerate the development of novel PNIs for use in emerging bioelectronic medicine therapies. To successfully adopt the methodology, the functional product delivery (design and fabrication) and testing (verification and validation) from each *Sprint* should be accelerated. This will permit the development team to learn about the product’s performance in a realistic end-user environment and allow consideration of the end-user’s experience in the use of the product. The identified barriers of the need for increased testing and the lack of comprehensive documentation for FDA review must also be overcome.

### 6.1 Accelerating sprints

#### 6.1.1 Design process

Reviewing existing PNIs provides insights into design considerations and could accelerate the design of new PNIs. The authors of several review articles (Ghafoor et al., 2017; Giagka and Serdijn, 2018; Russell et al., 2019; Wu and Peng, 2019; Cho et al., 2020; Larson and Meng, 2020; Wu and Guo, 2020; Yildiz et al., 2020; del Valle et al., 2021; Eiber et al., 2021; Farina et al., 2021; Paggi et al., 2021; Selim et al., 2021) provide different perspectives but a convergent view on primary design requirements for PNI. The three primary requirements are identifying the target nerve for interfacing, choosing the electrode type for interfacing, and determining the electronic hardware to stimulate or record neural activity. The other components of PNIs include electronics packaging and its connections with electrodes. Here, we focus our discussion on the development of neural electrodes.

Considerations for identifying the target nerve for interfacing with the electrode depend on the intended use of the proposed medical device. The selection of the electrode type (extraneural vs. intraneural) depends on the trade-off between the electrode performance properties (selectivity and specificity) versus invasiveness. The selectivity of the electrode (Ottestad and Orlovich, 2020) is defined as the ability to interface with a distinct group of nerve fibers. The specificity (Overstreet et al., 2019; Verma et al., 2023) is defined as the ability to activate a specific targeted group of nerve fibers to elicit a desired neural function. All the reviews cited above suggest that intrafascicular (intraneural) electrodes can achieve higher selectivity and specificity but at the cost of invasiveness.

The material considerations for fabricating electrodes depend on the stimulation and/or recording function of the PNI (Cogan, 2008; Grill et al., 2009). For stimulating, the electrode material should have sufficient charge-carrying capacity to stimulate and/or block neural activity without causing electrode material dissolution or neural tissue damage. For recording, the distance between the electrode and neural signal source and the impedance of the electrode should be chosen such that the signal-to-noise ratio is high.

#### 6.1.2 Device fabrication

3D printing, emerging bioprinting technologies, and off-the-shelf electronic or mechanical components can be used to fabricate functional devices to accelerate the verification and validation processes (Pena et al., 2017; Shepherd et al., 2018; Larson and Meng, 2020). This approach enables the development team to modify the designs quickly to conduct verification/validation studies. For example, biocompatible material was utilized to 3D print implantable components to verify and validate a quick to implant intrafascicular electrode (Q-PINE) array (Strauss et al., 2020), and a direct laser writing technique was used to print a nanoclip electrode capable of interfacing with ~50 μm diameter nerves (Lissandrello et al., 2017; Otchy et al., 2020).

#### 6.1.3 Verification process

Depending on the proposed medical device’s intended use for a specific PNI, one or more *in-vitro* and bench-top testing procedures in Table 1 could accelerate the verification process. In many instances, various jigs/fixtures may be needed for conducting verification tests. Instead of using conventional machining tools to build the jigs/fixtures, a 3D printer could offer a quick fabrication turnaround. 3D-printed fixtures are easier to scale if the device needs to be verified for varied sizes. In some cases, digital twins, i.e., computational models of the device (Morrison et al., 2018; Ahmed et al., 2023), can be used for verification. These testing procedures could also be used to verify the mechanical (e.g., electronics package hermicity) and electrical performances (e.g., communication protocols, power delivery) of the stimulating/recording electronics and electrode leads.

**Table 1:** The most common *in-vitro* tests utilized during the development of neural electrodes are listed (Cogan, 2008; Shepherd et al., 2018; Larson and Meng, 2020).

	Functional outcomes	Outcome measures
**Electrode performance verification tests**		
Electrochemical Impedance Spectroscopy in saline	Electrode stability Electrode recording capability	Impedance @ 1KHz for low impedance electrodes (<50KΩ) Full spectrum impedance and phase for high-impedance electrodes
Cyclic voltammetry	Charge storage capacity	Voltammogram Water window (visual detection of microscopic bubble formation)
Pulse testing/Voltage transients	Estimate the range of parameters for stimulation	Estimate maximum charge injection capacity per phase
**Electrode failure tests**		
Accelerated aging by soaking the electrodes passively in saline at varying temperatures starting from body temperature	Electrode corrosion Insulation breakdown	Impedance @ 1KHZ Electrochemical Impedance Spectroscopy Cyclic voltammetry High-resolution imaging
Cyclic mechanical fatigue testing	Electrode breakage Insulation breakage	Impedance @ 1KHZ Electrochemical Impedance Spectroscopy Cyclic voltammetry High-resolution imaging Video analysis

#### 6.1.4 Validation process

*Ex-vivo* studies in animal tissue followed by human cadaver tissue provide a fast turnaround for evaluating working prototypes in a real-world surgical environment, accelerating the validation process (Shepherd et al., 2018; Gupta et al., 2020). Multiple types of electrodes have been tested in porcine models because of the comparable neuroanatomy to humans (Shepherd et al., 2018; Trevathan et al., 2019; Settell et al., 2020; Strauss et al., 2020), allowing testing of scaled-up prototypes for human use. *Ex-vivo* animal studies can provide preliminary but critical validation data on several performance metrics that include but are not limited to passive electrical (impedance) performances, tethering forces on the leads connected to the stimulation/recording electronics package (Pena et al., 2017), and surgical planning of the implantation process. After *ex-vivo* studies, *in-vivo* animal studies can further reduce inadvertent and unknown risks.

### 6.2 Overcoming barriers

#### 6.2.1 Regulatory and ethical compliance during the validation process

Regulatory and ethical compliance in accelerating the validation process can be achieved by choosing an appropriate living tissue or simulated environment and following the research institute’s/company’s regulatory/ethical compliance procedures. The living tissue environment can be simulated using *ex-vivo* animal cadaver tissue sourced from other terminal *in-vivo* studies or an abattoir. These simulated but realistic environments help in the verification and validation process with a limited regulatory burden and provide valuable feedback to the development team. Following *ex-vivo* cadaver studies in appropriately sized tissue samples, it may be prudent to do studies in human cadaver tissue to validate the product further.

Following *ex-vivo* studies, *in-vivo* animal studies can be performed to evaluate the biofunctionality and biocompatibility of the device. The principles of the three R’s (Replace, Reduce, and Refine) can guide the developers in choosing the animal models with suitable anatomy and physiology needed for testing the device’s intended uses in *in-vivo* studies.

#### 6.2.2 Comprehensive documentation for regulatory approval

Regular and systematic reviews are conducted in the Waterfall methodology to generate comprehensive documentation. The same process can be implemented at the end of each *Sprint* for compiling documentation required by the FDA (Antonini et al., 2021; Slattery et al., 2022). In addition, documentation generated during the verification/validation process using digital twins or physical models can be submitted to the FDA through the Pre-Submisson/Q-Sub program to ensure appropriate validation is conducted with the final product, thereby accelerating the overall product development timeline.

## 7 Discussion

Navigating the complex medical device development (MDD) pathway (Guan et al., 2017; FDA, 2021; Lottes et al., 2022; Ashfaq, 2023), which involves translating innovative ideas into medical products, can be a challenging and resource-intensive task. Using the traditional Waterfall methodology can be slow and costly because the development team plans extensively and spends considerable upfront resources to make a fully functional product before releasing it to the users for testing. Further, if any unanticipated changes occur during testing (validation studies), then the development of the device has to revert to the beginning stage or product development has to be abandoned. Utilizing the Agile methodology can help accelerate the MDD pathway by delivering a verified product quicker for the users to test and for the product development team to revise iteratively, leading to cost reduction and faster delivery, ultimately benefiting patients in need. The enabling factors for adopting the Agile methodology for MDD include accelerating the verification and validation processes utilizing 3D printing and food-grade tissue for *ex-vivo* validation studies.

A review of hybrid design control methodologies (Schlauderer et al., 2015; Papadakis and Tsironis, 2020; Reiff and Schlegel, 2022; Slattery et al., 2022) such as hybrid V-model, hybrid Waterfall-Agile methodology, and a tailored Agile framework (Antonini et al., 2021), and white papers (Ashfaq, 2023) can provide insights into best practices for integrating the methodologies into existing new product introduction processes, and to overcome the barriers of managing regulatory compliance and approval. In addition, implementing the Agile methodology would foster the development and use of novel alternate methods (NAMs: *in chemico, in vitro, and in silico*) for the verification/validation processes, which could potentially reduce the use of animal models in biomedical research. Ultimately, the approach best suited for developing medical device hardware involving animal and human testing will depend on the verification and validation requirements as well as ethical and regulatory requirements. Further, emerging technologies, such as DevSecOps, which incorporates security and increased collaboration between the development team and operations, can be implemented for continued medical device development beyond the product’s initial release.

In conclusion, the Agile methodology with iterative verification and validation cycles across the design features can significantly improve de-risking a medical device’s clinical translation path by refining the design specifications to achieve the device’s intended use.

## Author contributions

AT: Conceptualization, Writing – original draft, Writing – review & editing. RJ: Funding acquisition, Supervision, Writing – review & editing, Conceptualization, Resources.
